# CPI-613 rewires lipid metabolism to enhance pancreatic cancer apoptosis via the AMPK-ACC signaling

**DOI:** 10.1186/s13046-020-01579-x

**Published:** 2020-04-28

**Authors:** Lixia Gao, Zhigang Xu, Zheng Huang, Yan Tang, Donglin Yang, Jiuhong Huang, Leilei He, Manran Liu, Zhongzhu Chen, Yong Teng

**Affiliations:** 1 National & Local Joint Engineering Research Center of Targeted and Innovative Therapeutics, College of Pharmacy, Chongqing University of Arts and Sciences, Chongqing, 402160 People’s Republic of China; 2 Department of Oral Biology and Diagnostic Sciences, Dental College of Georgia, Augusta University, 1120 15th Street, Augusta, GA 30912 USA; 3 Key Laboratory of Laboratory Medical Diagnostics Designated By Chinese Ministry of Education, Chongqing Medical University, Chongqing, 400016 China; 4 Georgia Cancer Center, Department of Biochemistry and Molecular Biology, Medical College of Georgia, Augusta University, Augusta, GA 30912 USA

**Keywords:** CPI-613, Pancreatic cancer, The AMPK-ACC signaling, Lipid metabolism, Apoptosis

## Abstract

**Background:**

Pancreatic cancer remains one of the most rapidly progressive and deadly malignancies worldwide. Current treatment regimens only result in small improvements in overall survival for patients with this cancer type. CPI-613 (Devimistat), a novel lipoate analog inhibiting mitochondrial metabolism, shows the new hope for pancreatic cancer treatment as an efficient and well-tolerated therapeutic option treated alone or in combination with chemotherapy.

**Methods:**

Pancreatic cancer cells growing in planar 2D cultures and 3D scaffold were used as research platforms. Cell viability was measured by MTT and alamarBlue, and apoptosis was assessed by JC-1 staining and flow cytometry with Annexin V-FITC/PI staining. The mechanism behind CPI-613 action was analyzed by western blot, transmission electron microscopy, and lipolysis assay kits, in the presence or absence of additional signaling pathway inhibitors or gene modifications.

**Results:**

CPI-613 exhibits anticancer activity in pancreatic cancer cells by triggering ROS-associated apoptosis, which is accompanied by increased autophagy and repressed lipid metabolism through activating the AMPK signaling. Intriguingly, ACC, the key enzyme modulating lipid metabolism, is identified as a vital target of CPI-613, which is inactivated in an AMPK-dependent manner and influences apoptotic process upon CPI-613. Blockade or enhancement of autophagic process does not increase or blunt apoptosis to CPI-613, but inhibition of the AMPK-ACC signaling significantly attenuates apoptosis induced by CPI-613, suggesting CPI-613-mediated lipid metabolism reduction contributes to its cytotoxicity in pancreatic cancer cells.

**Conclusions:**

These findings explore the critical role of lipid metabolism in apoptosis, providing new insights into the AMPK-ACC signaling axis in crosstalk between lipid metabolism and apoptosis in CPI-613 treatment.

## Background

Pancreatic cancer is the fourth most common cause of cancer mortality and recognized as the “king of cancers” in the world [1, 2]. This deadly disease is dependent on mitochondrial function for enhanced survival and aggressiveness, which is extremely difficult to detect in the early stages because of frequently few symptoms and lacking effective diagnosis. Despite significant improvements in clinical managements over the past two decades, the 5-year survival rate for pancreatic cancer patients remains lower than 10% [1–4]. Currently, the medical treatments are FOLFIRINOX (a four-drug combination of fluorouracil, leucovorin, irinotecan, and oxaliplatin) and gemcitabine plus nab-paclitaxel (G-nab), which provide a median overall survival of 11.1 months and 8.5 months, respectively [5, 6]. However, these treatments have moderately toxic effects and are often used to treat pancreatic cancer patients with good performance status. Therefore, safe and effective anticancer drugs are urgently needed in order to significantly prolong patients’ survival.

Lipoic acids, are mostly synthesized within the mitochondria as a cofactor necessary during mitochondrial energy metabolism, which have been shown to decrease cell viability and proliferation in pancreatic, breast, colon, ovarian, and lung cancer cells [7, 8]. CPI-613 (Devimistat) is the first member of a large set of analogs of lipoic acids, which strongly induces tumor repression by changing mitochondrial enzyme activity and redox status [9, 10]. CPI-613 is used as an inhibitor of mitochondrial tricarboxylic acid (TCA) for cancer treatment, because it can specifically target pyruvate dehydrogenase (PDH) and alpha-ketoglutarate dehydrogenase (α-KGDH) involved in the TCA cycle [11, 12]. The anticancer activity of CPI-613 has been confirmed against human pancreatic cancer in xenograft models with low side-effect toxicity [13]. A Phase I study reported there was a 61% objective response rate (including a 17% complete response rate) for metastatic pancreatic cancer patients receiving combination of CPI-613 with modified FOLFIRINOX (mFFX) [10]. A Phase III open-label trial to evaluate efficacy and safety of CPI-613 combined with mFFX versus FFX in patients with metastatic pancreatic cancer are now undergoing [14]. Nevertheless, the underlying molecular mechanisms of CPI-613 remain to be determined.

In this study, we show for the first time that the 5′ AMP-activated protein kinase (AMPK)-Acetyl-coenzyme A carboxylase (ACC) signaling is deeply involved in CPI-613-induced apoptosis in pancreatic cancer. Mechanistically, CPI-613 activates AMPK in pancreatic cancer cells, which in turn triggers autophagy and ACC inhibition. Interestingly, autophagy only marginally affects CPI-613-induced apoptosis. It appears that AMPK-dependent ACC inhibition contributes to reduced lipid metabolism upon CPI-613, which augments reactive oxygen species (ROS)-associated apoptosis in pancreatic cancer cells. These observations reveal that CPI-613 rewires lipid metabolism to enhance pancreatic cancer apoptosis via the AMPK-ACC signaling, providing new insights into the crosstalk between lipid metabolism reprogramming and apoptosis in cancer treatment.

## Methods

### Cell lines and culture

Human pancreatic cancer cell lines AsPC-1 and PANC-1 were purchased from the American Type Culture Collection (ATCC, Rockville, MD), and cultured in RPMI1640 medium containing 10% fetal bovine serum (FBS) at 37 °C in a humidified incubator supplied with 5% CO_2_.

### Reagents, antibodies, and standard assays

CPI-613 was obtained from Selleckchem (Houston, TX). 2′,7′-dichlorofluorescin diacetate (DCFH-DA), chloroquine (CQ), N-acetylcysteine (NAC), Compound C, simvastatin, and 5-(tetradecyloxy)-2-furoic acid (TOFA) were purchased from Sigma-Aldrich (St Louis, MO). Fatty acid and lipid metabolism antibody sampler kit and glycolysis antibody sampler kit were purchased from Cell Signaling Technology (Beverly, MA). Fatty acid and lipid metabolism antibody sampler kit includes antibodies against Acetyl-CoA Carboxylase (ACC), p-Acetyl-CoA Carboxylase (p-ACC) (Ser79), AceCS1, ACSL1, Lipin 1, ATP-Citrate Lyase, p-ATP-Citrate Lyase, Fatty Acid Synthase (FAS). Glycolysis antibody sampler kit includes GAPDH, PDH, HKI, HKII, LDHA, PKM2, PKM1/2, and PFKP. Antibodies against c-Caspase 3, PARP, p-mTOR (Ser2448), mTOR, p62, LC3B, p-AMPKα (Thr172), AMPKα, p-ULK1, ULK1, Bax and Bcl-2 were purchased from Cell Signaling. Apoptotic rate was determined using Annexin V-FITC Apoptosis Detection Kit (BD Biosciences, San Jose, CA). All flow cytometry data was analyzed using FlowJo software (Tree Star, Ashland, OR). Cell viability was determined by MTT assay, crystal violet staining, and alamarBlue Cell Viability Reagent (Thermo Fisher Scientific, Waltham, MA). Western blot, plasmid transfection and lentiviral infection were carried out as we previously described [15–17].

### Gene knockdown

Lentiviral-shRNA against ULK1 was obtained from GeneCopoeia (Rockville, MD), and the stable ULK1 knockdown AsPC-1 cells were generated using Lenti-Pac™ FIV Expression Packaging Kit (GeneCopoeia) according to the manufacturer’s instructions. Non-target shRNA (shNC) was used as a negative control in this study and the knockdown effect was confirmed by Western blot.

### JC-1 analysis for mitochondrial membrane potential (MMP)

MMP was measured by the JC-1 fluorescent probe (Invitrogen, Carlsbad, CA). CPI-613-treated or non-treated cells were incubated with JC-1 (1:1000 dilution) for 20 min at 37 °C. After PBS washing, cells were observed under a fluorescence microscope with the red fluorescence (550 nm excitation/600 nm emission) and green fluorescence channels (485 nm excitation/535 nm emission). Quantitative analysis of Red/Green fluorescence ratio was measured by NIH ImageJ software.

### Measurement of ROS levels

Intracellular ROS production was determined using the oxidant-sensing fluorescent probe DCFH-DA. Briefly, cells were incubated with 10 μM of DCFH-DA for 20 min at 37 °C and images were captured using a fluorescence microscope (IX-71, Olympus Corp., Tokyo, Japan). Median fluorescence intensity from at least 100 cells in randomly selected fields were quantified by NIH Image J software as we previously described [18, 19].

### Confocal laser scanning by high content analysis

The tandem labeled mRFP-GFP-LC3B plasmid was purchased from GeneCopoeia (Rockville, MD). In brief, cells were seeded into a 6-well plate overnight and transiently transfected with mRFP-GFP-LC3B using Lipofectamine 2000 according to the manufacturer’s instructions. After 48 h of transfection, cells were reseeded in CellCarrier 96-well microplates (PerkinElmer) in the presence or absence of 200 μM CPI-613. After a 24-h treatment, the fluorescent autophagy marker mRFP-GFP-LC3B was observed using a confocal laser mode (PerkinElmer, USA). The average number of mRFP-GFP-LC3B dots per cell was determined from three independent experiments. At least ten random fields representing 200 cells were counted in each well.

### Transmission electron microscopy (TEM)

Approximately 1.0 × 10^7^ cells treated with 200 μM CPI-613 or vehicle were fixed with 2% glutaraldehyde in 0.1 M sodium cacodylate (NaCAC) buffer (pH 7.4) for 45 min. The samples were post-fixed in 2% osmium tetroxide in NaCAC, stained with 2% uranyl acetate, dehydrated with a graded ethanol series and embedded in Epon-Araldite resin. Thin sections were cut with a Leica EM UC6 ultramicrotome (Leica Microsystems), collected on copper grids, and stained with uranyl acetate and lead citrate. Cells were observed in a Hitachi HT7700 transmission electron microscope and imaged with an UltraScan 4000 CCD camera and First Light Digital Camera Controller (Gatan).

### Three-dimensional (3D) cell culture

Briefly, 1 × 10^5^ cells were seeded into 48-well SeedEZ scaffold (Lena Bioscience, Atlanta, GA) supplied with complete medium. After 3 days of culture, cells growing in the SeedEZ scaffold were treated with 200 μM CPI-613 for 5 days, and cell viability was measured by alamarBlue at 545/590 nm ex/em, followed by phalloidin staining and imaging as we previously described [20].

### Lipolysis analysis

Lipid droplets and free fatty acids (FFA) released into the culture medium of pancreatic cancer cells were measured to evaluate lipolysis. AsPC-1 and PANC-1 cells were treated with 200 μM CPI-613 for 48 h prior to lipolysis assessment. To determine lipid droplets, cells were fixed with 4% paraformaldehyde and stained with the dye Oil-Red-O (Sigma-Aldrich) for 30 min using the isopropanol method, followed by processed for haematoxylin staining. The released FFA levels were measured by Free Fatty Acid Quantification Kit (Abcam, Cambridge, UK) according to the manufacturer’s instruction. The absorbance at 570 nm was measured immediately afterwards on a microplate reader.

### Statistical analysis

Statistical analyses were performed by unpaired Student’s t test for two group comparisons and one-way analysis of variance (ANOVA) for multi-group comparisons at a significance level of *p* < 0.05. Data were presented as means ± SD from three or more independent experiments.

## Results

### CPI-613 alters mitochondrial morphology and inhibits cell viability in pancreatic cancer cells

**Fig. 1:**
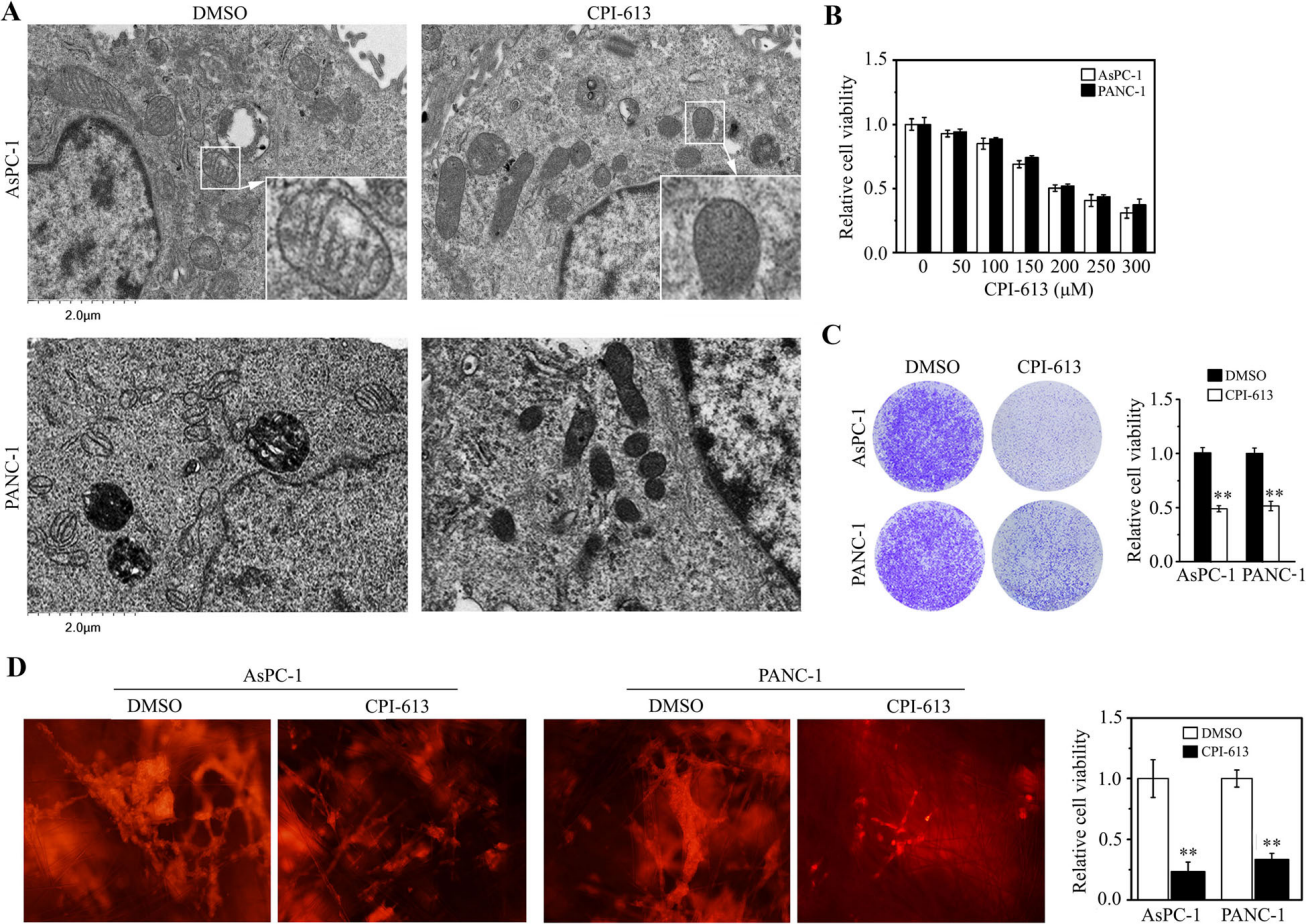
CPI-613 induces the morphological changes of mitochondrial and inhibits cell viability in pancreatic cancer cells. **a** The effects of CPI-613 on mitochondrial morphology in AsPC-1 and PANC-1 cells determined by TEM. **b**, **c** The effects of CPI-613 on cell viability. **b** AsPC-1 and PANC-1 cells were treated with a dose range of CPI-613 for 48 h, followed by MTT assays. **c** AsPC-1 and PANC-1 cells were treated with or without 200 μM CPI-613 for 7 days, followed by crystal violet staining and quantification by a plate reader at OD570. **d** The effects of CPI-613 on 3D cell viability. AsPC-1 and PANC-1 cells growing in the SeedEZ scaffold were treated with or without 200 μM CPI-613 for 5 days, and cell viability was measured by Phalloidin staining (left) and alamarBlue (right), respectively. **p < 0.01

To understand whether mitochondrial morphological alterations are responsive to CPI-613, we examined the phenotype of mitochondria in AsPC-1 cells, in the presence or absence of CPI-613. TEM analysis showed that CPI-613 dramatically reduced mitochondrial cristae junctions and overall cristae morphology (Fig. 1a). The same phenotype of mitochondria was seen in CPI-613-treated PANC-1 cells (Fig. 1a), indicating that CPI-613 is potent to disrupt mitochondrial structure of pancreatic cancer cells. To determine the effective dose of CPI-613 in pancreatic cancer cells, AsPC-1 and PANC-1 cells were treated with CPI-613 at different concentrations ranging from 50 μM to 300 μM for 48 h. MTT assay showed that CPI-613 exhibited inhibitory effects on cell proliferation dose-dependently (Fig. 1b), which was confirmed by crystal violet staining even after a long-term culture (Fig. 1c). The IC50 of CPI-613 was ~ 200 μM in both AsPC-1 and PANC-1 cells (Fig. 1b), and this dose was used in the following experiments. 3D culture systems take advantages of reliable experimental results, simple, and convenient operation in the evaluation of drug responses due to truly simulating the microenvironment [20]. We then seeded AsPC-1 and PANC-1 cells in the SeedEZ 3D scaffold and treated with 200 μM CPI-613 for 5 days. In line with the data from 2D cultures, CPI-613 dramatically decreased cell viability in SeedEZ (Fig. 1d), suggesting it also exhibits strong cell-killing effects in 3D cultures.

### CPI-613 induces ROS-associated apoptosis in pancreatic cancer cells

**Fig. 2:**
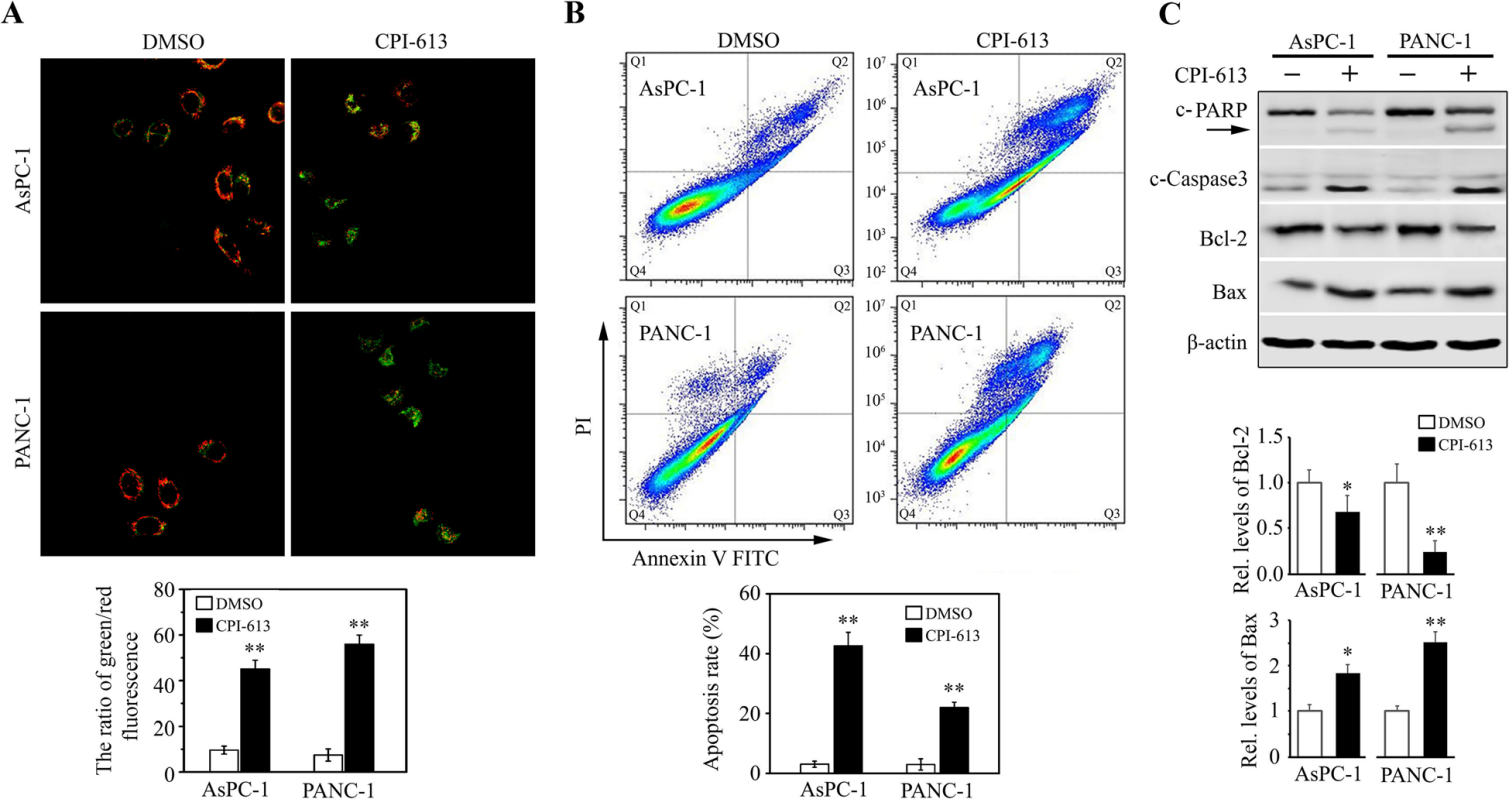
CPI-613 induces apoptosis in pancreatic cancer cells. **a**, **b** The effects of CPI-613 on apoptosis. AsPC-1 and PANC-1 cells were treated with or without 200 μM CPI-613 for 48 h, and apoptosis was determined by flow cytometry with Annexin V-FITC/PI staining (**a**) and JC-1 fluorescent probe (**b**), respectively. **c** The molecular changes in apoptosis determined by Western blot with antibodies against PARP, c-Caspase 3, Bcl-2, and Bax. The quantification of Bcl-2 and Bax protein levels from three independent experiments was shown in lower panel. *p < 0.05; **p < 0.01

To study the mechanisms underlying the cytotoxicity of CPI-613 in pancreatic cancer cells, we determined apoptosis of AsPC-1 and PANC-1 cells by JC-1 staining assay in the presence or absence of CPI-613. This analysis showed that most of DMSO-treated cells were clearly red, but the increase in green fluorescence and the concomitant disappearance of red fluorescence were observed in cells treated with CPI-613 (Fig. 2a), indicating that CPI-613 has the ability to induce MMP dissipation. To further assess apoptotic rate upon drug treatment, flow cytometry analysis was performed after FITC Annexin V/ PI staining, which showed CPI-613 increased apoptosis up to 40% in AsPC-1 and 20% PANC-1 cells (Fig. 2b). In addition, there were increased levels of cleaved PARP, cleaved Caspase 3, and Bax in both cell lines treated with CPI-613, coupled with decreased levels of Bcl-2 (Fig. 2c). These data indicate that CPI-613 can effectively induce apoptosis in pancreatic cancer cells.

**Fig. 3:**
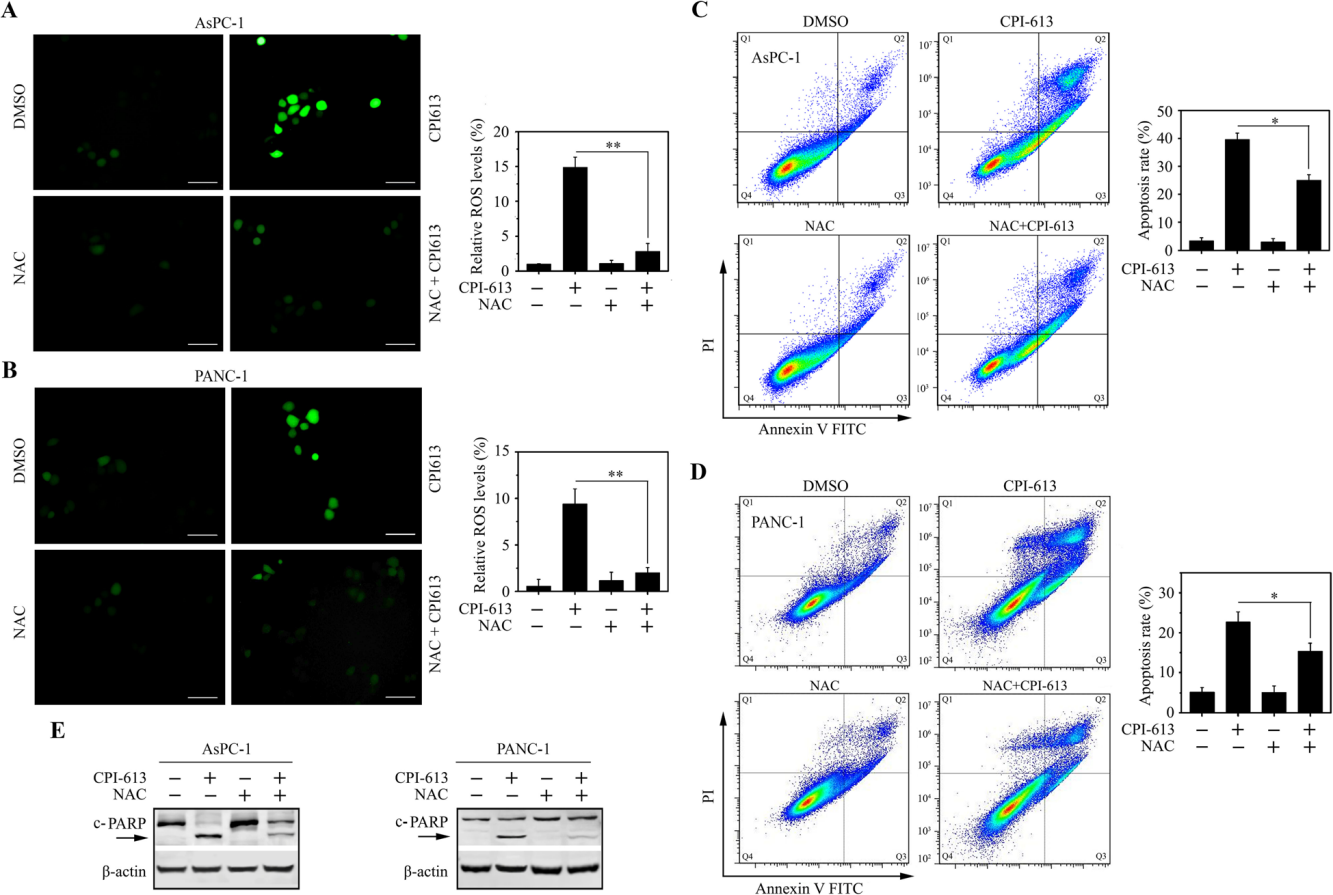
CPI-613-induced apoptosis is associated with increased ROS levels in pancreatic cancer cells. **a**, **b** The effects of NAC on CPI-613-induced ROS generation. AsPC-1 (a) and PANC-1 (**b**) cells were treated with or without 200 μM CPI-613 for 12 h in the presence or absence of 5 mM NAC, and ROS levels were determined and quantitated by DCFH-DA staining. **c**, **d** The effects of NAC on CPI-613-induced apoptosis. AsPC-1 (**c**) and PANC-1 (**d**) cells were treated with or without 200 μM CPI-613 for 48 h in the presence or absence of 5 mM NAC, and apoptosis was determined by flow cytometry with Annexin V-FITC/PI staining. **e** The molecular changes in apoptosis were determined by Western blot with antibody against PARP. *p < 0.05; **p < 0.01

Elevated intracellular levels of ROS induces oxidative stress, leading to cell death [21]. To determine whether CPI-613-induced apoptosis was associated with ROS alterations, the DCFH-DA fluorescent dye was used to detect their levels. This assay showed a significant increase of fluorescence in both AsPC-1 and PANC-1 cells following CPI-613 treatment (Fig. 3a and b). We next treated these cells with or without NAC, a potent antioxidant as the scavenger of ROS, in the presence of CPI-613. The addition of NAC significantly attenuated the increase of ROS levels in CPI-613 treatment (Fig. 3c and d), alleviating CPI-613-induced apoptosis as indicated by a reduction in the levels of cleaved PARP in CPI-613-treated cells (Fig. 3e). These observations support the notion that CPI-613 promotes apoptosis through accumulating ROS in pancreatic cancer cells.

### CPI-613 triggers the AMPK-ULK1 autophagic pathway in pancreatic cancer cells

**Fig. 4:**
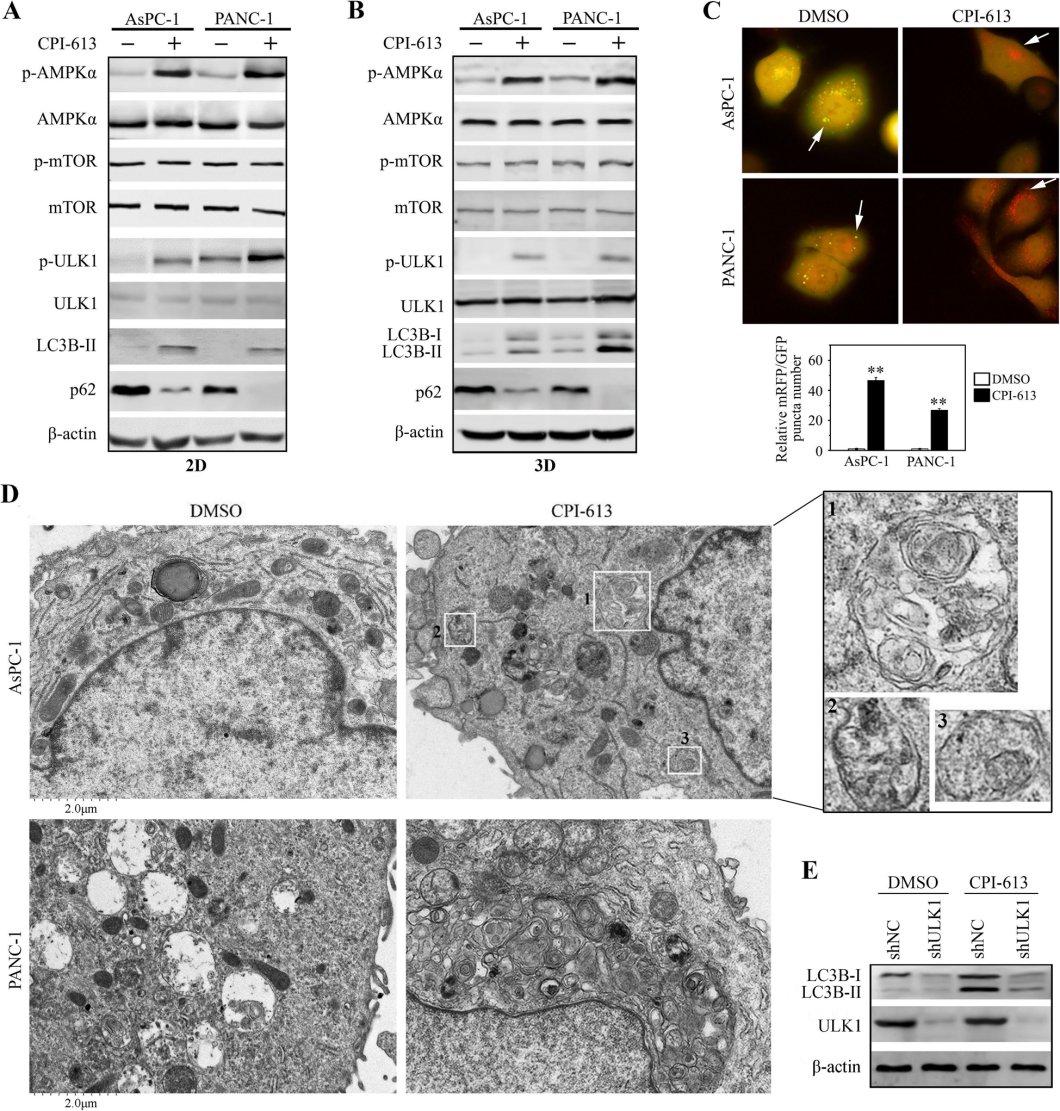
CPI-613 promotes the AMPK-ULK1 autophagic signaling in pancreatic cancer cells. **a**, **b** The effects of CPI-613 on AMPK-dependent autophagic signaling. AsPC-1 and PANC-1 cells growing in 2D culture dishes (**a**) or the SeedEZ 3D scaffold (**b**) were treated with or without 200 μM CPI-613 for 48 h, followed by Western blot with the indicated antibodies. **c**, **d** The effects of CPI-613 on autophagy. AsPC-1 and PANC-1 cells were transfected with mRFP-GFP-LC3B plasmid and treated with or without 200 μM CPI-613 for 48 h, followed by fluorescence analysis and quantification of LC3B-positive autophagosomes (**c**). **d** The effects of CPI-613 on autophagy determined by TEM. AsPC-1 and PANC-1 cells were treated with or without 200 μM CPI-613 for 48 h and subjected to TEM analysis. Autophagosomes in CPI-613-treated AsPC-1 cells were indicated in boxes. **e** The effects of ULK1 knockdown (shULK1) on LC3B-II under the influence of CPI-613. **p < 0.01

Apoptosis and autophagy can be stimulated by the same stresses [22]. As the part of our investigation, we found that increased accumulation of LC3B-II, an effective marker of autophagic flux, in CPI-613-treated AsPC-1 and PANC-1 cells (Fig. 4a). Along with this change, another autophagy marker, p62, was decreased (Fig. 4a). This was the same observed in CPI-613-treated cells growing in the SeedEZ scaffold (Fig. 4b). To verify the effect of CPI-613 on autophagy, we transfected mRFP-GFP-LC3B plasmid into these cells and observed the changes of autophagosomes puncta number by imaging the green fluorescent signal shifting to red. Both AsPC-1 and PANC-1 cells underwent autophagy following CPI-613 treatment, as evidenced by a significant increase in LC3B-positive puncta compared with control cells (Fig. 4c). TEM further revealed numerous double-membraned vacuoles in CPI-613-treated cells that contained fragments of the endoplasmic reticulum and other cytoplasmic components (Fig. 4d). Collectively, these data indicate that CPI-613 is a potent inducer of autophagy in pancreatic cancer cells.

AMPK contributes to efficient autophagosome maturation and lysosomal fusion, prompting us to examine the changes in AMPK activation in cells treated with CPI-613 or not. As shown in Fig. 4a, the phosphorylation levels of AMPK were increased in both AsPC-1 and PANC-1 cells following CPI-613 treatment. The AMPK-mTOR pathway commonly participate in autophagic process. However, there was no noticeable change in phospho-mTOR levels in the presence or absence of CPI-613 (Fig. 4a and b), suggesting CPI-613 may regulate autophagy through other pathways. We then determined the status of ULK1, the major downstream molecule of AMPK in autophagic signaling, in cells treated with CPI-613 or not. The phosphorylation levels of ULK1 were markedly increased along with the increase of phospho-AMPK in CPI-613-treated cells compared with control cells (Fig. 4a and b). We then depleted ULK1 in AsPC-1 cells using the lentivirus mediated shRNA, which showed knockdown of ULK1 dramatically attenuated the accumulation of LC3B-II form following CPI-613 treatment (Fig. 4e), further supporting a role of ULK1 in CPI-613-induced autophagy.

### Autophagy does not crosstalk with apoptosis in pancreatic cancer cells upon CPI-613

**Fig. 5:**
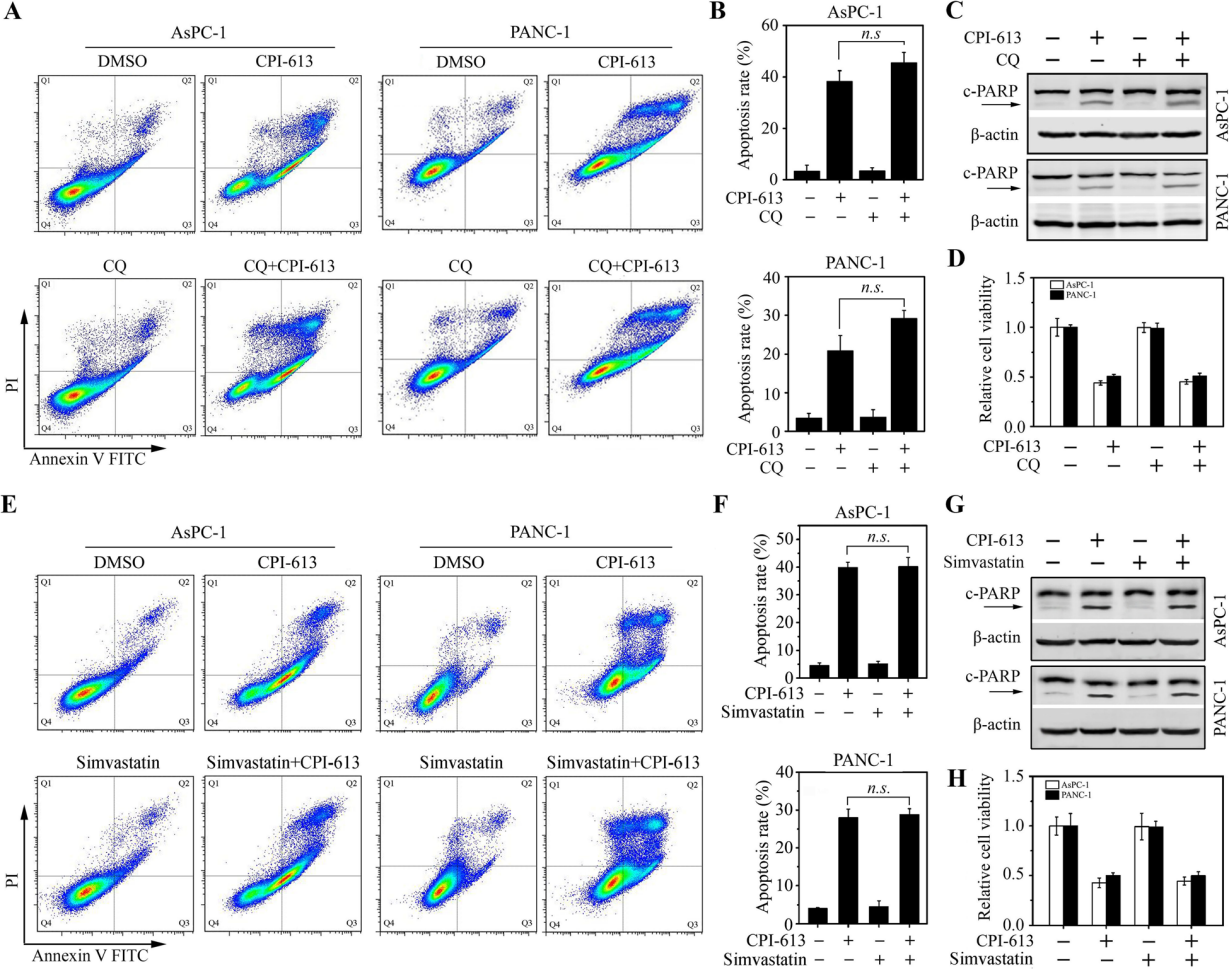
Blockade or enhancement of autophagy has a marginal effect on CPI-613-induced apoptosis in pancreatic cancer cells. **a**-**d** The effects of the autophagy inhibitor CQ on CPI-613-induced apoptosis. AsPC-1 and PANC-1 cells were treated with or without 200 μM CPI-613 for 48 h in the presence or absence of 20 μM CQ, and apoptosis was determined by flow cytometry with Annexin V-FITC/PI staining (**a**, **b**) and Western blot with antibody against PARP (**c**). **d** The effects of CQ on CPI-613-induced cell viability determined by MTT assays. **e**-**h** The effects of the autophagy inducer simvastatin on CPI-613-induced apoptosis. AsPC-1 and PANC-1 cells were treated with or without 200 μM CPI-613 for 48 h in the presence or absence of 10 μM simvastatin, and apoptosis was determined by flow cytometry with Annexin V-FITC/PI staining (**e**, **f**) and Western blot with antibody against PARP (**g**). **h** The effects of simvastatin on CPI-613-induced cell viability determined by MTT assays. The representative results of flow cytometry and quantitative data from three independent experiments were shown in (**a**, **e**) and (**b**, **f**), respectively. *n.s.*: not significant

CQ is an effective autophagy inhibitor blocking lysosomal degradation [23]. To explore the functional relationship between CPI-613-induced autophagy and apoptosis, AsPC-1 and PANC-1 cells were treated with 20 μM CQ in the presence or absence of CPI-613, followed by apoptosis measurement. CQ treatment alone had no noticeable effect on cell apoptosis and viability in both cell lines (Fig. 5). Moreover, CQ failed to alter the apoptosis levels induced by CPI-613, as evidenced by no changes in apoptotic rate and the c-PARP levels in cells treated with CPI-613 alone or combined with CQ (Fig. 5a-c). In line with these data, additional CQ in CPI-613 treatment did not rescue or decrease cell viability during CPI-613 treatment (Fig. 5d). To confirm the results, AsPC-1 and PANC-1 cells were co-treated with CPI-613 and an autophagy activator simvastatin. In the presence of CPI-613, no significant changes in apoptotic rate (Fig. 5e-g) and cell viability (Fig. 5h) were observed in cells treated with 10 μM simvastatin or not. Taken together, these data strongly suggest that autophagy does not contribute to CPI-613-induced apoptosis in pancreatic cancer cells.

### CPI-613 suppresses lipid metabolism in pancreatic cancer cells by AMPK-dependent ACC inhibition

**Fig. 6:**
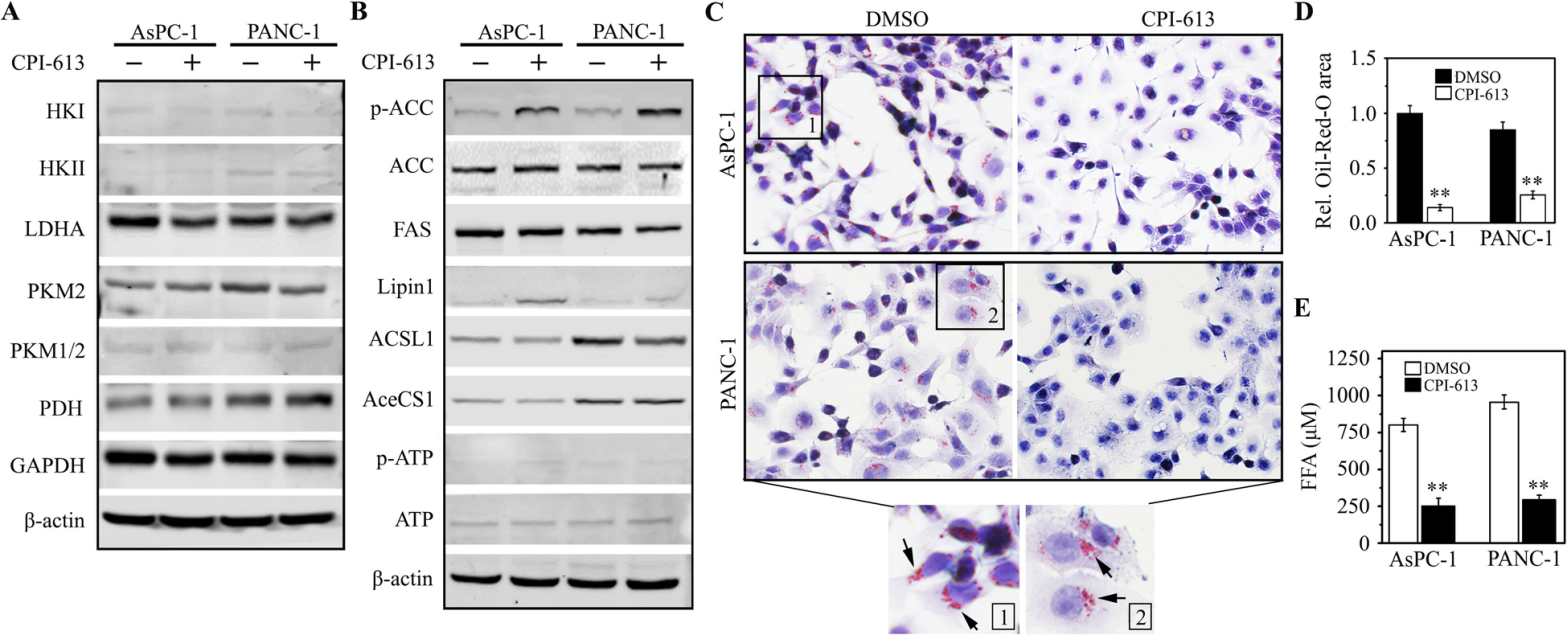
CPI-613 inhibits lipid metabolism through suppressing ACC activity in pancreatic cancer cells. **a**, **b** The effects of CPI-613 on lipid metabolism and glycolysis. AsPC-1 and PANC-1 cells were treated with or without 200 μM CPI-613 for 48 h, followed by Western blot with Glycolysis antibody sampler kit (**a**) and Fatty acid and lipid metabolism antibody sampler kit (**b**), respectively. **c**, **d** The effects of CPI-613 on the accumulation of lipid droplets determined by Oil-Red-O staining. The representative images and quantitative data from three independent experiments were shown in (**c**) and (**d**), respectively. **e** The effects of CPI-613 on released FAA levels evaluated by Free Fatty Acid Quantification Kit. **p < 0.01

AMPK is the key regulator in cellular energy sensing and restoration of metabolic balance [24]. To further elucidate the role of AMPK signaling in drug action of CPI-613, we determined the changes in metabolism-associated molecules in pancreatic cancer cells with or without CPI-613 treatment. These molecular alterations were screened by Western blot with Glycolysis antibody sampler kit and Fatty acid and lipid metabolism antibody sampler kit. This analysis revealed there was only a marginal effect of CPI-613 on the glycolytic pathway (Fig. 6a). ACC is a substrate of AMPK providing the malonyl-CoA substrate for the biosynthesis of fatty acids, which is switched off by phosphorylation and activated by dephosphorylation [25]. Interestingly, the phosphorylation levels of ACC were remarkably increased in either AsPC-1 or PANC-1 cells treated with CPI-613 (Fig. 6b), suggesting the possible function of CPI-613 in downregulating lipogenesis by inhibiting ACC activity. To validate this, we stained AsPC-1 and PANC-1 cells with the Oil-Red-O dye. As depicted in Fig. 6c, lipid droplets became evident in these cells without CPI-613 treatment. In contrast, decreased lipid accumulation was seen in CPI-613-treated cells (Fig. 6c and d), which was accompanied by reduced FFA levels in the culture medium (Fig. 6e). These findings indicate that CPI-613 potently suppresses lipid metabolism in pancreatic cancer cells, which is mainly through inactivating ACC via AMPK.

### The AMPK-ACC signaling contributes to CPI-613-induced apoptosis in pancreatic cancer cells

**Fig. 7:**
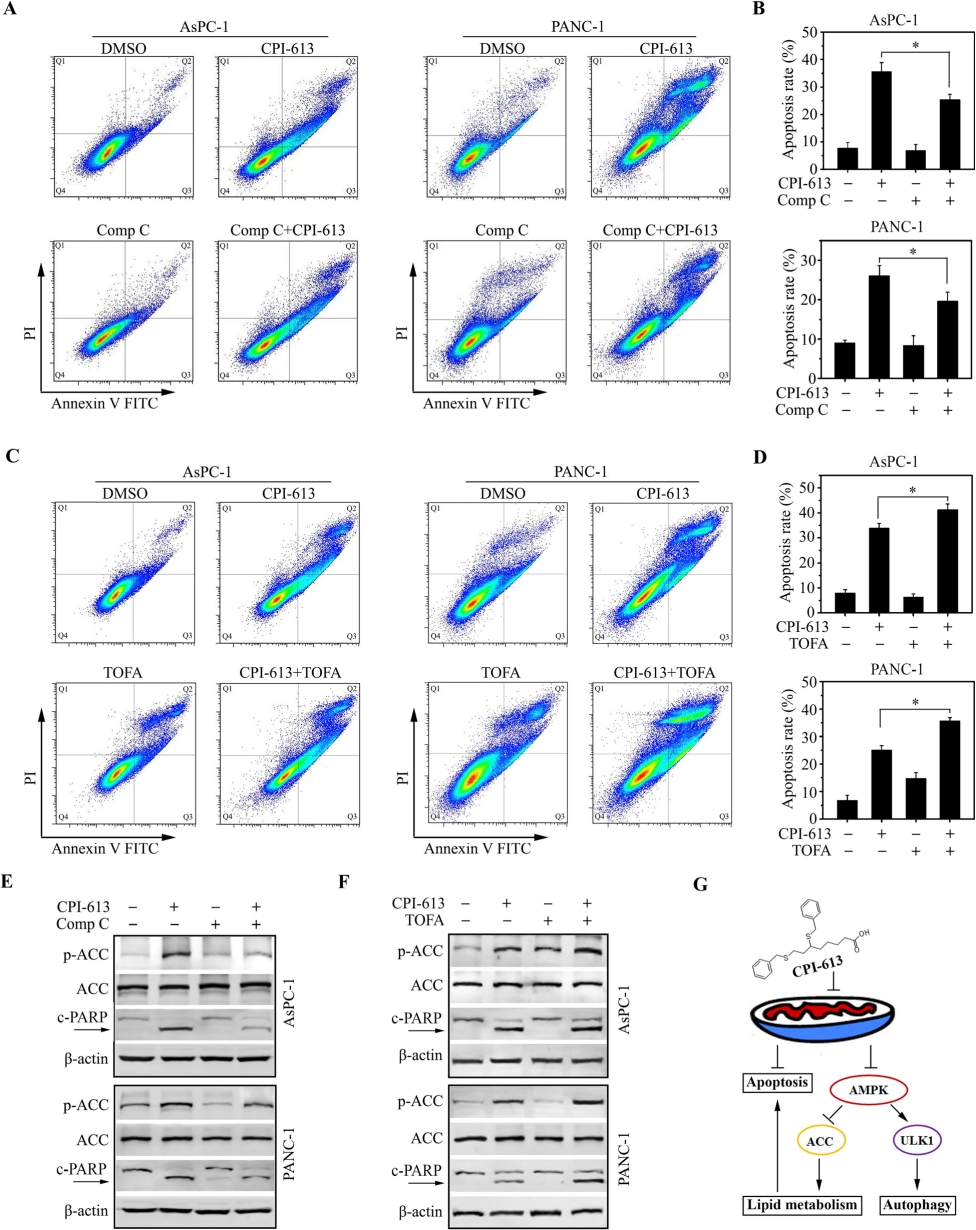
The AMPK-ACC signaling contributes to CPI-613-induced apoptosis in pancreatic cancer cells. **a**, **b**, **e** The effects of AMPK inhibitor Compound C on CPI-613-induced apoptosis. AsPC-1 and PANC-1 cells were treated 10 μM Compound C for 1 h, followed by treated with 200 μM CPI-613 for 48 h. Apoptosis was determined by flow cytometry with Annexin V-FITC/PI staining (**a, b**) and western blot with antibodies against PARP and p-ACC (**e**). **c**, **d**, **f** The effects of ACC inhibitor TOFA on CPI-613-induced apoptosis. AsPC-1 and PANC-1 cells were treated with 10 μM TOFA for 1 h, followed by treated with 200 μM CPI-613 for 48 h. Apoptosis was determined by flow cytometry with Annexin V-FITC/PI staining (**c, d**) and Western blot with antibodies against PARP and p-ACC (**f**). The representative results of flow cytometry and quantitative data from three independent experiments were shown in (**a**, **c**) and (**b**, **d**), respectively. (**g**) Schematic representation of the mechanism of CPI-613 action in pancreatic cancer cells. *p < 0.05

We next studied the importance of AMPK-ACC signaling in CPI-613-induced apoptosis. Our data showed pretreatment of the AMPK inhibitor Compound C attenuated increased ACC phosphorylation in CPI-613 treatment, which also significantly alleviated CPI-613-induced apoptosis in both AsPC-1 and PANC-1 cells (Fig. 7a, b and e). TOFA can be converted to 5-tetradecyloxy-2-furoyl-CoA (TOFyl-CoA) and exerts an allosteric inhibition on ACC [26], which was used to treat AsPC-1 and PANC-1 cells together with CPI-613. Increased apoptosis coupled with enhanced ACC phosphorylation were observed in cells receiving combination treatment compared with each drug alone (Fig. 7c, d and f), suggesting that apoptosis promoted by CPI-613 is highly dependent on the AMPK-ACC signaling in pancreatic cancer cells.

## Discussion

Pancreatic cancer has an exceedingly poor prognosis with a 5-year survival compared with many other solid tumors [1–4]. Current strategies do not target genetic features of pancreatic cancer and patients with this type of cancer have few therapeutic options [2–4]. CPI-613, a novel lipoate analog with the function inhibiting mitochondrial metabolism, has been reported to produce strong tumor growth inhibition with little or no side-effect toxicity at 25 mg/kg or even higher therapeutic doses [9]. Further clinical Phase I study reveals the maximum tolerated dose of CPI-613 was 500 mg/m^2^ in pancreatic cancer patients enrolled between 2013 and 2016 [10]. The median number of treatment cycles given at the maximum tolerated dose was 11, and the median follow-up of patients treated at the maximum tolerated dose was 378 days [10]. This study also provides encouraging evidence that the novel treatment modality of CPI-613 in combination with mFFX chemotherapy was safe and well tolerated in patients with metastatic pancreatic cancer [10]. Although a Phase III clinical trial of this treatment has been designed and is undergoing [14], the underlying molecular mechanism remains unknown. We report here that CPI-613 exhibits strong anticancer activity in pancreatic cancer cells via ROS-associated apoptosis, which is coupled with AMPK activation. Upon CPI-613, the upregulated AMPK-ACC signaling rewires lipid metabolism, promoting the progression of apoptosis in pancreatic cancer cells. This novel mechanism explores the critical role of AMPK-dependent ACC inhibition in CPI-613-induced apoptosis, adding a new layer to understand the crosstalk between lipid metabolism and apoptosis in cancer treatment (Fig. 7g). Thus, our study may shed light on CPI-613-based treatment to lead to desired effect in pancreatic cancer patients.

The TCA cycle-mediated generation of ROS is a key mediator for cell survival. CPI-613 has been reported to selectively target the altered form of mitochondrial TCA in tumor cells, leading to apoptosis via changes in mitochondrial enzyme activities and redox status [11]. This is true in pancreatic cancer cells as we provide the evidence that CPI-613-induced apoptosis is tightly associated with ROS. Inhibiting ROS in these cells using the antioxidant NAC can prevent cell from apoptosis in CPI-613 treatment. Most interestingly, CPI-613-induced apoptosis is also regulated by the AMPK signaling. While mTOR and ULK1 are two well-reported targets of AMPK that can be co-activated by AMPK and subsequently trigger autophagy [15, 22], our findings unravel that autophagy triggered by CPI-613 only lies in the AMPK-ULK1 signaling.

Autophagy is a self-protective response in living cells or organisms to adapt to stress and extracellular cues [15, 22]. The mutual relationship between autophagy and apoptosis is highly context-dependent. A majority of cases, it seems that apoptosis and autophagy are mutually inhibitory, although there is accumulating evidence showing autophagy tends to be pro-apoptotic rather than anti-apoptotic in some particular conditions [22, 27]. The observations from the present study suggest that AMPK-dependent autophagy only has a marginal effect on CPI-613-induced apoptosis in pancreatic cancer cells, which prompted us to study the other possible role of AMPK in CPI-613 treatment as it is considered to be an important component controlling many other pathways.

Lipid metabolism participates in the regulation of many cellular processes (e.g. cell survival and apoptosis) and its disorder is pathologically linked to cancer [28]. ACC as the rate-limiting enzyme in fatty acid synthesis, plays a pivotal role in the growth and viability of cancer cells [29]. Phosphorylation by AMPK converts ACC to an inactive form, leading to the reduction in lipid metabolism. In this study, we explore that CPI-613 is potent to increase the ACC phosphorylation levels through activating the AMPK signaling in pancreatic cancer cells. We also reveal that inactivation of either AMPK or ACC attenuates CPI-613-induced apoptosis, suggesting the deep involvement of the AMPK-ACC signaling in treatment-associated apoptosis. Although the results from pancreatic cancer cell lines are sufficient to prove the role and importance of AMPK-ACC signaling in CPI-613 treatment, the current study did not include the in vivo confirmation. It would be interesting to determine whether this molecular mechanism is highly relevant to the therapeutic outcomes of CPI-613 in preclinical cancer models. Moreover, whether ROS affects the AMPK-ACC signaling axis and how ACC-mediated lipid metabolism contributes to ROS-associated apoptosis, are warranted to better understand the drug action of CPI-613.

## Conclusions

Our findings explore the critical role of AMPK-dependent ACC inhibition in apoptosis and provide new insights into the crosstalk between lipid metabolism and apoptosis in CPI-613 treatment. The knowledge gained from this study should help us develop novel strategies to selectively push cancer cells toward terminate fates.

## Data Availability

All data generated or analyzed during this study are included in the manuscript.

## Abbreviations

3D: Three-dimensional
α-KGDH: Alpha-ketoglutarate dehydrogenase
ACC: Acetyl-coenzyme A carboxylase
AMPK: 5′ AMP-activated protein kinase
ANOVA: One-way analysis of variance
ATCC: The American Type Culture Collection
CQ: Chloroquine
DCFH-DA: 2′,7′-dichlorofluorescin diacetate
NaCAC: Sodium cacodylate
mFFX: Modified FOLFIRINOX
FAS: Fatty Acid Synthase
FBS: Fetal bovine serum
FFA: Free fatty acids
FOLFIRINOX: Fluorouracil, leucovorin, irinotecan, and oxaliplatin
G-nab: Gemcitabine plus nab-paclitaxel
NAC: N-acetylcysteine
PDH: Pyruvate dehydrogenase
ROS: Reactive oxygen species
TCA: Tricarboxylic acid
TEM: Transmission electron microscopy
TOFA: 5-(tetradecyloxy)-2-furoic acid
